# Impact of Multimorbidity Subgroups on the Health Care Use and Clinical Outcomes of Patients With Tuberculosis: A Population-Based Cohort Analysis

**DOI:** 10.3389/fpubh.2021.756717

**Published:** 2021-10-08

**Authors:** Qin Chen, Yang Che, Yue Xiao, Feng Jiang, Yanfei Chen, Jifang Zhou, Tianchi Yang

**Affiliations:** ^1^Hwa Mei Hospital, University of Chinese Academy of Sciences, Ningbo, China; ^2^Ningbo Municipal Center for Disease Control and Prevention, Institute of Tuberculosis Prevention and Control, Ningbo, China; ^3^School of International Pharmaceutical Business, China Pharmaceutical University, Nanjing, China

**Keywords:** tuberculosis, delivery of health care, health expenditures, multimorbidity—coordination of care—general practitioner, latent class analysis

## Abstract

**Background:** Multimorbidity is defined as the existence of two or more chronic health conditions in the same individual. While patients with tuberculosis commonly have multiple conditions at diagnosis, such as HIV, diabetes, and depression, to the authors' knowledge, there is limited information on the patterns of multimorbidity, and how the types and combinations of conditions could impact the healthcare utilization, expenditure, and TB outcomes.

**Methods:** An observational cohort study of adult patients diagnosed with tuberculosis was conducted using the Chinese Center for Disease Control and Prevention (CDC)'s National TB Information System (NTBIS) linked to the Ningbo Regional Health Care Database (NRHCD) (2015–2020). Latent class analysis was used to identify comorbidity groups among the subset with ≥2 conditions including TB. Group-level health care use, expenditure, and treatment outcomes were compared with patients without chronic conditions using multivariate regression models.

**Results:** A total of 9,651 patients with TB were identified, of whom approximately 61.4% had no chronic conditions, 17.4% had 1 chronic condition, and 21.3% had ≥2 chronic conditions. Among those with ≥1 chronic condition other than TB, 4 groups emerged: (1) general morbidity (54.4%); (2) cardiovascular morbidity without complications (34.7%); (3) cardiovascular morbidity with complications (5.0%); (4) respiratory morbidity (5.9%). The respiratory morbidity group experienced the highest expenditures, at 16,360 CNY more overall (95% CI, CNY 12,615–21,215) after adjustment compared with TB patients without chronic conditions. The respiratory morbidity and cardiovascular morbidity with complications group also had the lowest odds of favorable TB outcomes [adjusted odds ratio (aOR), 0.68; 95% CI, 0.49–0.93] and (aOR 0.59, 95% CI 0.42–0.83), respectively. The cardiovascular morbidity without complications group had the highest odds of successful TB treatment (aOR, 1.40; 95% CI, 1.15–1.71).

**Conclusions:** Multimorbidity is common among patients with TB. The current study identified four distinct comorbidity subgroups, all of which experienced high, yet differential, rates of health care use. These findings highlight the need for urgent reforms to transform current fragmented TB care delivery and improve access to other specialists and financial assistance.

## Introduction

Tuberculosis (TB) is a serious threat to global public health. The World Health Organization (WHO) has estimated that over 10.4 million people are infected by *Mycobacterium tuberculosis*, and that 1.67 million deaths are attributable to TB (1). Multimorbidity is defined as the co-existence of two or more chronic conditions within an individual (2). Due to the nature of chronic infection with TB, patients who are diagnosed with TB may already have chronic conditions such as HIV infection. The presence of multimorbidity has important implications on patient outcomes and healthcare costs.

Chronic communicable diseases such as HIV and hepatitis virus infection are common in TB patients. The risk of developing TB is significantly higher among patients living with HIV infection. Hepatitis B co-infection with TB is common in China, and hepatitis B virus surface antigen (HBsAg)-positive people account for over 7.8% of the population (3). Additionally, the ongoing COVID-19 pandemic is likely to disrupt TB management and lower TB treatment success rates. A recent meta-analysis on the first wave of COVID-19 patients has found that the prevalence of TB ranged between 0.47 and 4.47%, and TB infection was associated with more severe COVID-19 cases (4).

In addition to chronic communicable conditions, global TB eradication initiatives have been hampered by the increasing burden of non-communicable diseases (NCD), especially in low- and middle-income countries. Population aging, urbanization, and unhealthy lifestyles all contribute in a high comorbid disease pattern among patients with TB. A cross-sectional study based on a medical chart review found that ~1.14% of TB subjects had multimorbidity. Patients with multimorbidity tend to be older, female sex, and to have higher mortality risks (5). Nevertheless, the prevalence of multimorbidity could be underestimated due to poor record-keeping, lack of standardized multimorbidity classification methods, and limited sample size (6).

Traditionally, studies have focused on single conditions such as HIV co-infection, diabetes, and alcohol use disorders that affect the clinical outcomes of TB treatment. There is a need for additional information on chronic condition profiles, as well as care patterns and associated expenditures. Coordination of specialty care and primary services is essential for managing TB patients with multimorbidity. A better understanding of the comorbidity profile could facilitate early detection, improve care coordination, and inform the decision-making relating to patients at greater risk of unfavorable TB treatment outcomes. Until now, no study has compared clinical outcomes and healthcare expenditure in TB patients with varying degrees of multimorbidity.

The objective of this study was to identify and describe chronic morbidity groups among patients with tuberculosis in real-world settings, and to explore differences in demographic and clinical characteristics across subgroups. Finally, we aim to assess the impact of multimorbidity on healthcare utilization, expenditure, and clinical outcomes in real-world settings.

## Methods

### Study Design

We performed a population-based retrospective cohort study among patients with TB aged 18 years old or above in Ningbo city, in the People's Republic of China. Tuberculosis cases were confirmed based on the NTBIS. Patients whose diagnosis changed or with insufficient clinical information of healthcare utilization during the baseline period (1 year prior to TB diagnosis) or during the follow-up period were excluded. During the baseline period (1 year preceding their index date, which is the TB diagnosis date), important confounders, namely lifestyle, comorbidities, and TB-related characteristics were defined. The current study was approved by the Institutional Review Board (IRB) of Ningbo Municipal Center for Disease Control and Prevention and the requirement for informed consent was waived. The data were fully de-identified and sensitive information such as patients' names and national ID numbers were anonymized. We thus made re-identification impossible to safeguard patients' privacy. The quality of reporting in the included studies was assessed using the Strengthening the Reporting of Observational Studies in Epidemiology (STROBE) checklist (7), which are commonly used for reporting the quality of observational studies.

### Data Sources and Data Linkage

Ningbo is a coastal city in the eastern region of the People's Republic of China, with a population of nearly 8.2 million residents in 2018. Since 2005, Ningbo Health and Family Planning Commission initiated a Health Information System covering all healthcare facilities within its jurisdiction. Electronic health records from hospitals and community clinics were collected and integrated to form a comprehensive administrative healthcare database. All records are linked deterministically using the personal identity code. This code is unambiguously assigned to all Chinese citizens as well as registered foreign citizens. The linked records are deidentified before they are further processed and analyzed. By 2020, the system covered all 65 public hospitals and 154 community health centers or township health centers, including all 12 TB designated hospitals and TB centers of excellence (Ningbo Hwa Mei Hospital).

Information regarding subject lifestyle, vital signs, laboratory results, inpatient and outpatient records, and pharmacy use were available. The outpatient, inpatient, and pharmacy records comprised patient identity code, date of diagnosis, diagnosis name, and diagnosis code (International Classification of Diseases, Tenth Revision, ICD-10), as well as procedure codes, costs, and prescription dispensing details. The data content is updated on a daily basis.

The TB registry provides information on TB diagnosis, treatment, and outcomes. The TB registry records the TB patients' personal identity code, the date of diagnosis, TB clinical classification, and genetic determinants of drug resistance, sputum, and culture results at diagnosis and follow-up were recorded using a standard electronic form by trained staff. TB outcomes were ascertained using standard WHO outcomes definitions for tuberculosis: cure, treatment completion, treatment failure, death from any cause, default, and transfer out. We defined successful outcomes as cure or completion of treatment and unfavorable outcomes such as treatment failure or death. All death records including date of death and causes of death were further confirmed by linkage to the mortality database of the Ningbo Municipal Public Security Bureau.

### Cohort Description

**Figure 1:**
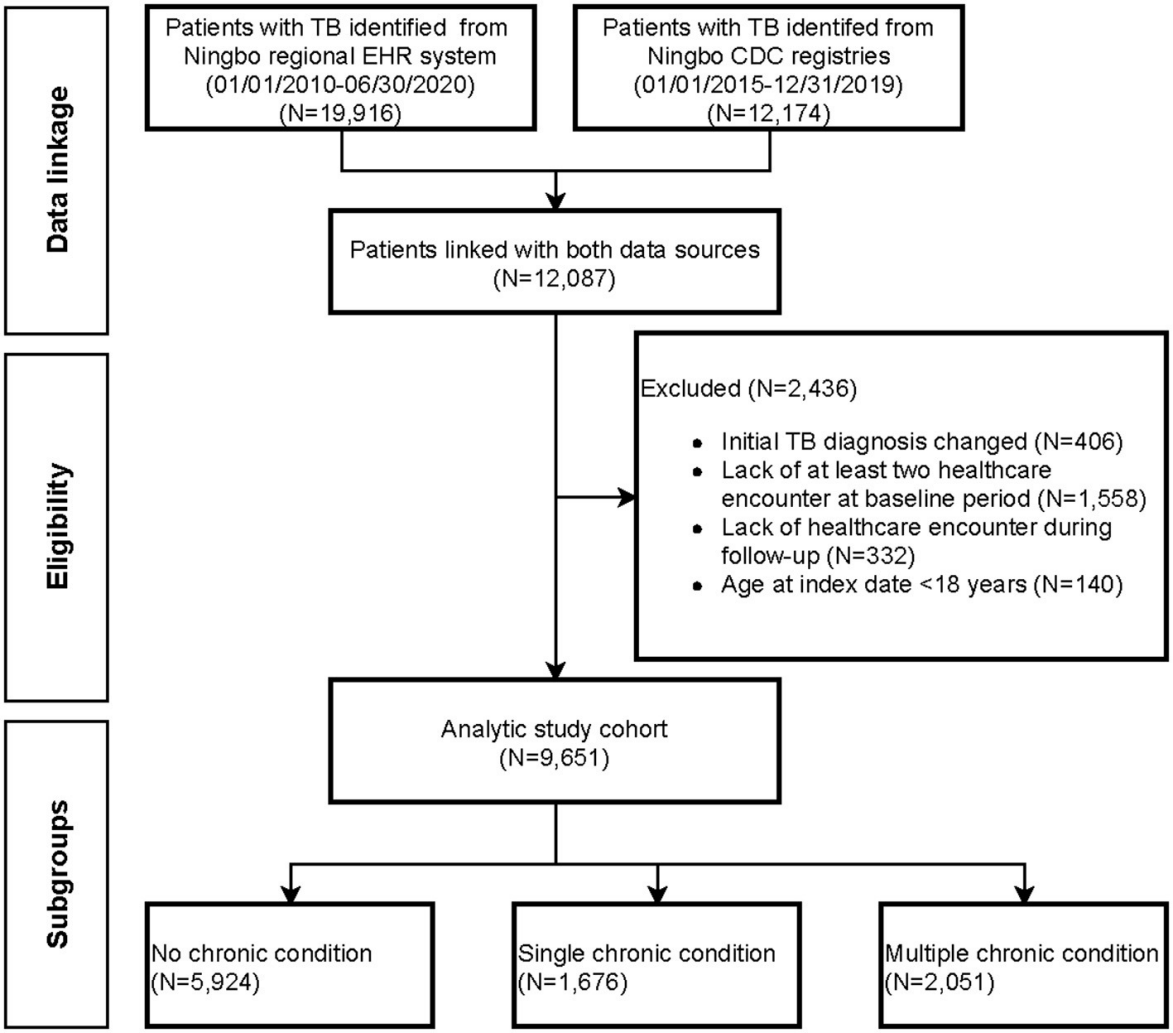
Patient flow diagram documenting the application of selection criteria to arrive at the final analytical cohort.

The cohort selection is shown in Figure 1. Patients were required to be aged ≥ 18 years at TB diagnosis. In addition, eligible subjects were required to have at least two healthcare encounters during the baseline period to ensure capturing of important demographic and clinical information prior to TB diagnosis. The reason for requiring eligible subjects to have at least two encounters is to exclude patients who are not continuously followed within the Ningbo health system, which could lead to underestimation of morbidity burdens. Subject characteristics, including chronic condition profiles, were measured over the baseline period. Subjects were followed up from date of TB diagnosis to date of treatment completion, death, end of study (30 June 2020), or loss to follow-up, whichever came first. Healthcare use and expenditure were measured in both the baseline period and follow-up period.

### Identification of Chronic Conditions and Generation of Morbidity Groups

ICD-10 diagnosis codes from all inpatient discharge summaries and outpatient encounters were collected and categorized using the Chronic Condition Indicator (CCI) algorithms (v2021.1) developed by Healthcare Cost and Utilization Project (HCUP) (8). The algorithms systematically scan all diagnosis fields and generated classification of distinct chronic conditions (9). Identified chronic conditions that appeared in two outpatient encounters on different dates or in 1 inpatient hospitalization during the baseline period were retained in the analysis. Patients were categorized as having 0, 1, or multiple (2 and above) chronic conditions, excluding TB.

Latent class analysis (LCA) was performed on the subset of patients with ≥2 chronic conditions. LCA is a data-driven, model-based clustering method that is capable of identifying underlying subgroups of patients with similar multimorbidity patterns. We included all chronic conditions with a prevalence of 1% and above. A series of models were developed and the final model was selected based on statistical convergence, the goodness of fit, interpretability, and parsimony (10–12). A subject-level latent class multimorbidity group was assigned based on the highest predicted probability of membership, calculated from individual chronic condition profiles (13).

### Variables

We measured four outcomes during the follow-up period: (1) hospital admission; (2) outpatient visit; (3) monthly total healthcare expenditures; and (4) monthly patient out-of-pocket payment. Total expenditures were calculated based on overall inpatient, outpatient and pharmacy expenditures from a healthcare system's perspective. Estimates were adjusted for inflation using the consumer price index (CPI) and presented in 2014 Chinese Yuans (¥). A hundred CNY was equivalent to 16.3 USD in 2014.

Additional demographic, lifestyle habits, insurance type, TB-related variables, prior resource use were measured during the baseline period. Demographic variables included age, sex, ethnicity, occupation, education level, and marital status.

### Statistical Analysis

Patients in each of the morbidity groups were compared with patients without pre-existing chronic conditions at baseline. Categorical variables were compared using chi-square tests and continuous variables were compared using non-parametric Kruskal-Wallis tests accounting for non-normal distribution of variables. Missing data were addressed using missing indicators.

We compared the utilization of inpatient services across latent chronic condition groups using a multivariate logistic lasso regression model, with excess risk for admission expressed in adjusted odds ratio (OR) and 95% Confidence intervals (CIs). We classified healthcare expenditure into 3 types: outpatient expenditure, inpatient expenditure, and outpatient pharmacy expenditure. To account for excess zeros and the skewed non-zero distribution nature of healthcare expenditure data, we used a 2-part regression model to identify the association between morbidity groups and healthcare expenditure. We first fitted a logistic model to estimate the association between morbidity groups with none vs. any utilization of healthcare. Among patients with non-zero expenditure, a second model of the generalized linear model (GLM) using gamma distribution with a log link function (14, 15). We used GLM in the second stage rather than the ordinary least squares of log-transformed healthcare expenditure because the former relaxes the homoscedastic error assumption, which is essential for retransformation to the raw scale (16).

Analyses were conducted using SAS 9.4 statistical software (SAS Institute Inc., Cary, NC, USA), and open-source statistical software R 4.0.3 (poLCA package, https://CRAN.R-project.org). Two-side *P* < 0.05 was considered as statistically significant.

## Results

### Sample Description

A total of 9,651 eligible patients with TB were included in the present cohort study, the patient flow diagram is shown in Figure 1. The final analytical cohort consisted of 3,073 (31.8%) females, with a mean age of 48.8 years (SD, 18.7) (Table 1). Approximately 30.5% of patients were aged above 60 years and 4,128 (42.8%) patients were covered by urban employee basic medical insurance schemes at the time of TB diagnosis. The prevalence of HIV infection was 0.31% and the majority had newly registered TB. Pre-existing chronic conditions were observed in 38.6% of TB patients. The average number of chronic conditions was 1.02 (SD< 1.99), and 2,051 (21.3%) had ≥2 chronic conditions. Essential primary hypertension was the most common chronic condition (14.3%), followed by hyperlipidemia (4.0%), chronic obstructive pulmonary disease (COPD) (2.2%), and atherosclerotic heart disease (2.2%).

**Table 1:** Baseline characteristics of tuberculosis patients cohort (*N* = 9,651).

**Characteristics**	***N***	**%**
**Age**		
Mean age at TB diagnosis (SD), y	48.8	18.7
Median age (IQR), y	49	32–63
**Age group, y**		
18–40	3,641	37.7
41–50	1,465	15.2
51–60	1,603	16.6
61–70	1,570	16.3
71+	1,372	14.2
**Female sex**	3,073	31.8
**Insurance type**		
UEBMI	4,128	42.8
URRMI	771	8.0
Other	4,752	49.2
**Retreatment status**		
Newly diagnosed	8,998	93.2
Retreatment	653	6.8
**TB clinical types**		
III	8,998	93.2
II	653	6.8
IV	8,758	90.7
I	54	0.6
Extra pulmonary TB	795	8.2
**HIV positive**	30	0.31
**Chronic conditions**		
Mean, SD	1.02	1.99
None	5,924	61.4
1	1,676	17.4
2+	2,051	21.3
**Charlson comorbidity index**		
0	3,152	32.7
1	1,389	14.4
2	1,314	13.6
3	1,323	13.7
4+	2,473	25.6

*TB, tuberculosis; SD, standard deviation; IQR, interquartile range; UEBMI, urban employee basic medical insurance; URRMI, urban and rural resident medical insurance; HIV, human immunodeficiency virus*.

### Identification of Morbidity Groups

The LCA of patients with at least one chronic condition (*N* = 3,727) identified 4 morbidity subgroups. Demographic and clinical characteristics are presented in Table 2. The general morbidity group accounted for the largest percentage of TB patients with chronic conditions (54.5%), with an average of 2.5 chronic conditions. The mean age of patients in this group was 54.5 years (SD, 16.3), which is the youngest among all latent class subgroups. The most prevalent chronic condition in this group was chronic viral hepatitis B infection (8.62%) and chronic pharyngitis (5.57%). The cardiovascular morbidity without complications group (34.7% of the current study cohort) experienced a high prevalence of hypertension (85.6%) and hyperlipidemia (20.3%). The mean age was 65.2 years (SD, 12.1 years). The cardiovascular morbidity with complications group (5.0% of the current study group) was among the oldest groups, with a mean age of 74.5 years (SD, 10.5 years) at the time of TB diagnosis. Patients in this group experienced primarily hypertension (85.9%), hyperlipidemia (59.5%), atherosclerotic heart disease (53.0%), and heart failure (42.2%). In this group, the percentage of patients with mental health conditions was the highest (4.3%). Finally, the respiratory morbidity subgroup accounted for 220 (5.9%) of patients, and was predominantly male (83.6%). The mean age was 73.6 years (SD, 10.5 years) and had the highest percentage of patients with retreatment status (15.5%). The most prevalent condition was chronic obstructive pulmonary disease (80.5%) and essential primary hypertension (31.4%).

**Table 2:** Characteristics of model-driven chronic condition groups among eligible patients with tuberculosis.

	**Respiratory morbidity**		**Cardiovascular morbidity with complications**		**Cardiovascular morbidity without complications**		**General morbidity**		***P-*value**
**Class name**									
Sample size	220	5.9%	185	5.0%	1,292	34.7%	2,030	54.5%	–
Mean age (SD)	73.6	10.5	74.5	10.5	65.2	12.1	51.4	16.3	<0.0001
**Age group**									
18–40	2	0.9%	0	0.0%	34	2.6%	558	27.5%	<0.0001
41–50	3	1.4%	3	1.6%	109	8.4%	368	18.1%	
51–60	12	5.5%	20	10.8%	269	20.8%	465	22.9%	
61–70	60	27.3%	43	23.2%	444	34.4%	376	18.5%	
71+	143	65.0%	119	64.3%	436	33.7%	263	13.0%	
**Female sex**	36	16.4%	51	27.6%	409	31.7%	673	33.2%	<0.0001
**Insurance type**									
UEBMI	114	51.8%	64	34.6%	635	49.1%	875	43.1%	<0.0001
URRMI	8	3.6%	19	10.3%	80	6.2%	149	7.3%	
Other	98	44.5%	102	55.1%	577	44.7%	1,006	49.6%	
**Retreatment status**	34	15.5%	24	13.0%	91	7.0%	166	8.2%	<0.0001
**Smoking status**									
Current Smoker	16	7.3%	22	11.9%	139	10.8%	156	7.7%	<0.0001
Former smoker	28	12.7%	19	10.3%	113	8.7%	89	4.4%	
No smoker	89	40.5%	69	37.3%	521	40.3%	715	35.2%	
Unknown	87	39.5%	75	40.5%	519	40.2%	1,070	52.7%	
**Alcohol consumption status**									
Drinker	18	8.2%	10	5.4%	68	5.3%	81	4.0%	<0.0001
Non-drinker	115	52.3%	99	53.5%	691	53.5%	864	42.6%	
Unknown	87	39.5%	76	41.1%	533	41.3%	1,085	53.4%	
**Chronic conditions**									
Mean, SD	4.0	4.0	8.6	8.5	3.0	2.6	2.5	2.0	<0.0001
Atherosclerotic heart disease	24	10.9%	98	53.0%	87	6.7%	0	0.0%	<0.0001
Cardiac arrhythmia	28	12.7%	40	21.6%	82	6.3%	13	0.6%	<0.0001
Chronic obstructive pulmonary disease	177	80.5%	36	19.5%	2	0.2%	0	0.0%	<0.0001
Chronic pharyngitis	5	2.3%	4	2.2%	48	3.7%	113	5.6%	0.0098
Chronic viral hepatitis B infection	1	0.5%	7	3.8%	13	1.0%	175	8.6%	<0.0001
Emphysema	44	20.0%	8	4.3%	0	0.0%	101	5.0%	<0.0001
Essential primary hypertension	69	31.4%	159	85.9%	1,106	85.6%	43	2.1%	<0.0001
Gastric ulcer	7	3.2%	22	11.9%	36	2.8%	50	2.5%	<0.0001
Gout	0	0.0%	23	12.4%	50	3.9%	39	1.9%	<0.0001
Heart failure	46	20.9%	78	42.2%	12	0.9%	0	0.0%	<0.0001
Hyper lipidemia	8	3.6%	110	59.5%	262	20.3%	10	0.5%	<0.0001
Non-toxic single thyroid nodule	6	2.7%	26	14.1%	42	3.3%	88	4.3%	<0.0001
Other disorders of glycoprotein metabolism	12	5.5%	42	22.7%	17	1.3%	95	4.7%	<0.0001
Sarcoidosis of lung	4	1.8%	11	5.9%	20	1.5%	88	4.3%	<0.0001
Sequelae of respiratory and unspecified tuberculosis	15	6.8%	21	11.4%	2	0.2%	100	4.9%	<0.0001
Presence of ≥1 mental health condition	2	0.9%	8	4.3%	27	2.1%	51	2.5%	0.1261

*SD, standard deviation; UEBMI, urban employee basic medical insurance; URRMI, urban and rural resident medical insurance scheme*.

### Association of Morbidity Groups With TB Treatment Outcome

As shown in Table 3, a total of 8,182 (84.8%) had successful TB treatment outcomes, with 29.6% cured and 55.2% completing TB treatment. The overall treatment success rate was higher in patients with no chronic conditions (87%), compared with morbidity subgroups. The cardiovascular morbidity without complications group and general morbidity group had overall TB treatment success rates of 82.5 and 82.8%, respectively. Cardiovascular morbidity with complications had the lowest TB treatment success rate of 65.9%, while 71.8% of the patients in the respiratory morbidity group had favorable outcomes overall.

**Table 3:** Association between chronic condition groups and clinical outcomes.

**Outcomes**	**Overall**		**No chronic** **conditions**		**Respiratory** **morbidity**		**Cardiovascular morbidity** **with complications**		**Cardiovascular morbidity** **without complications**		**General** **morbidity**	
Sample size	9,651		5,924		220		185		1,292		2,030	
Overall successful treatment	8,182	84.8%	5,155	87.0%	158	71.8%	122	65.9%	1,066	82.5%	1,681	82.8%
Cured	2,857	29.6%	1,660	28.0%	105	47.7%	52	28.1%	426	33.0%	614	30.2%
Treatment completed	5,325	55.2%	3,495	59.0%	53	24.1%	70	37.8%	640	49.5%	1,067	52.6%
MDR-TB	121	1.3%	69	1.2%	3	1.4%	1	0.5%	11	0.9%	37	1.8%
ADR	40	0.4%	10	0.2%	3	1.4%	0	0.0%	16	1.2%	11	0.5%
Loss to follow-up	945	9.8%	539	9.1%	27	12.3%	37	20.0%	139	10.8%	203	10.0%
Non-TB death	165	1.7%	47	0.8%	15	6.8%	19	10.3%	37	2.9%	47	2.3%
Treatment failure	150	1.6%	87	1.5%	5	2.3%	3	1.6%	15	1.2%	40	2.0%
TB death	48	0.5%	17	0.3%	9	4.1%	3	1.6%	8	0.6%	11	0.5%

*TB, tuberculosis; MDR-TB, multi-drug-resistant tuberculosis; ADR, adverse drug reaction*.

Among the types of unfavorable outcomes, 9.8% of patients were lost to follow-up and 2.2% died during TB treatment. Among the 213 patients who died, 48 death events were attributable to TB, and 165 (1.7%) died from causes other than TB. Approximately 1.3% of patients developed MDR-TB, the percentage of patients who developed MDR-TB were comparable across all subgroups.

After adjusting for age and sex, patients in the cardiovascular morbidity with complications group had a 41% (95% CI 0.46–0.77) lower odds for achieving successful TB treatment outcomes, when compared with patients without chronic conditions. Conversely, patients in the cardiovascular morbidity without complications group had a 27% (95% CI 1.10–1.47) higher odds for achieving successful TB treatment outcomes. After adjusting for additional insurance type, TB retreatment status, and lifestyle variables, patients in the respiratory morbidity group (OR 0.68, 95% CI 0.49–0.93) and cardiovascular morbidity with complications group (OR 0.59, 95% CI 0.42–0.83) were less likely to have favorable TB outcomes. On the other hand, patients from the cardiovascular morbidity without complications group were more likely to achieve successful outcomes (OR 1.40, 95% CI 1.15–1.71) (Supplementary Table 1).

### Health Resource Use Across Morbidity Groups

Overall healthcare expenditure was assessed from TB diagnosis date until TB treatment termination, death, end of the study period (June 30, 2020), or loss-to-follow-up, whichever came first. The univariate summary of overall healthcare expenditure is presented in Supplementary Table 2. On average, the all-cause total cost during TB treatment was 10,052 CNY (SD, 20,976 CNY, in 2014 terms). Differences were also observed with regard to latent class subgroups. Patients with no pre-existing chronic conditions experienced the lowest overall costs at 4,691 CNY, while patients from the cardiovascular morbidity with complications group had the highest expenditure of 19,839 CNY (SD, 40,835 CNY). The excessive healthcare spending in this group was driven by inpatient expenditure.

**Figure 2:**
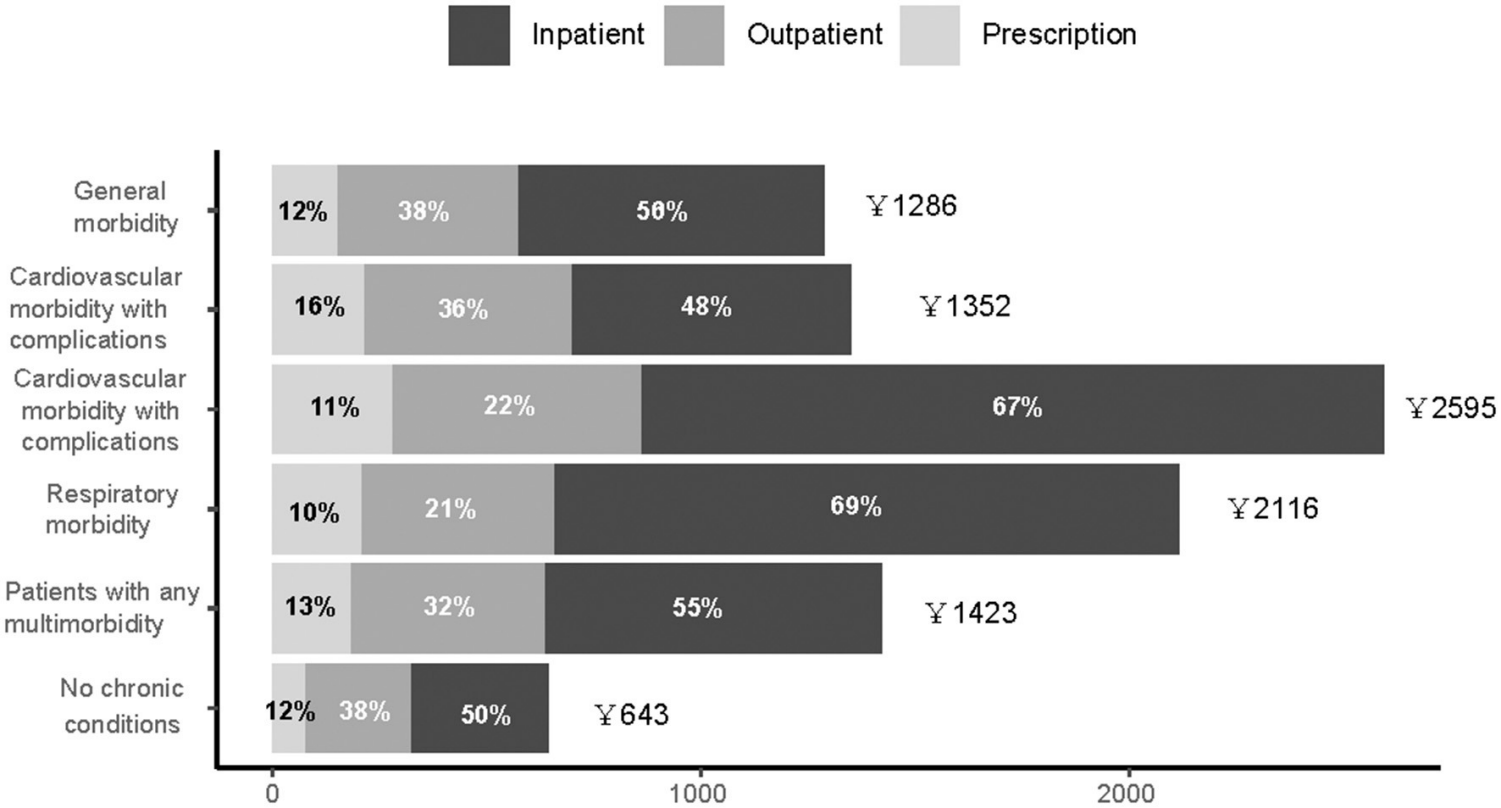
Distribution of average overall individual healthcare expenditures among patients with tuberculosis, stratified by types of services.

To account for differential treatment durations, individual monthly healthcare expenditures according to chronic condition groups are presented in Figure 2. Monthly average and median healthcare expenditures were higher in the cardiovascular morbidity with complications group, followed by the respiratory morbidity group. However, monthly out-of-pocket (OOP) expenditures ranged between 237 and 531 CNY, with the share of OOP payment ranging between 20.0 and 36.9% (Supplementary Table 3).

In multivariable analysis, as shown in Supplementary Table 4, after adjusting for relevant demographic and clinical characteristics, patients in the respiratory morbidity group on average incurred an additional of 16,360 CNY (95% CI 12,615–21,215), compared with patients without any chronic conditions. Patients in cardiovascular morbidity with and without complication groups had incremental costs of 10,987 CNY (95% CI 9,542–12,651 CNY) and 10,279 CNY (95% CI 8,999–11,741 CNY), respectively. Patients in the general morbidity group had lower overall expenditure when compared with the reference group (7,832 CNY, 95% CI 6,928–8,853 CNY).

Patients with chronic conditions incurred statistically significantly greater OOP expenditures. Over the course of TB treatment, patients in the respiratory morbidity group had higher expenditure (4,543 CNY, 95% CI 3,163–6,525 CNY), when compared to patients without chronic conditions. Patients in the general morbidity group had a lower adjusted increase in OOP expenditure, costing 3,136 CNY (95% CI 2,632–3,736 CNY) more than the non-chronic condition group (Supplementary Table 4).

## Discussion

In this retrospective cohort analysis of 9,651 patients with TB, there were three notable findings. First, older patients have higher rates of multimorbidity, compared to previous publications. Second, we identified four distinct latent subgroups based on concurrent diagnoses, and these groups are predictive of TB treatment outcomes. Third, healthcare utilization and expenditure were highly correlated with latent subgroups, thereby such findings are informative in identifying potential targets of continuous care quality improvement.

TB patients with multimorbidity accounted for ~40% of eligible subjects in this population-based cohort. We successfully linked a majority of patients from the National TB registry with healthcare encounter records. By examining over 10 years' regional electronic health records, we employed a systematic approach in identifying chronic conditions using validated algorithms (9). This method not only enabled us to characterize concurrent conditions at the time of TB diagnosis but also estimate the healthcare use patterns and associated expenditure from both healthcare systems and patients' perspectives. We found significant variability in both acute care utilization, outpatient services, and pharmaceutical products. While patients with cardiovascular conditions and respiratory morbidity experienced worse outcomes, these patients incurred substantially higher monthly overall and OOP expenditure. The TB treatment success rates were lower among patients with greater comorbidity burdens compared with patients without multimorbidity or within the general morbidity group.

Interestingly, we found that after adjusting for relevant covariates, patients in the cardiovascular morbidity without complications group had higher treatment success rates relative to the non-chronic condition group. A higher proportion of patients in this group achieved cured outcomes (33.0% vs. 28.0%), despite a higher morbidity burden and older age. On the other hand, we found that patients without any comorbid conditions were more likely to be younger patients of the floating population of migrant workers without health insurance coverage. We hypothesized that these patients from the cardiovascular morbidity without complications group were more likely to be covered by a more generous UEBMI scheme and living in Ningbo (17). Thus, patients from this subgroup experienced more continuous health services, allowing for more swift care transitions (18).

Another strength of this study is the use of LCA in providing information regarding clinically meaningful subgroups (19). More simplistic methods employed a fixed list of chronic conditions in defining chronic condition groups, our data-driven approach allowed inclusion of all observed diagnoses to identify distinct subgroups (20–22). This approach takes account for potentially joint comorbidity patterns between more severe chronic conditions such as heart failure and common illnesses like hypertension and gout, our study is capable of elucidating more detailed condition combinations. Although LCA has been widely used to identify clusters of patient-reported symptoms or trajectories of healthcare utilization, to the best of our knowledge, no prior study to date has examined multimorbidity in TB patients using a population-based, real-world dataset. We distinguished subgroups of cardiovascular morbidity with and without complications, has to our knowledge, not been previously reported. The complex comorbidity combinations in a sample subset of patients (respiratory morbidity group 5.9%, and cardiovascular morbidity with complications, 5.0%), with on average 4.0 and 8.6 concurrent chronic conditions warrant further investigation. Patients in this group experienced greater risks for treatment discontinuation due to loss-to-follow-up and death, suggestive of multispecialty care plan in meeting the needs of these patients.

While the overall prevalence of mental health disorders is low, we observed higher psychological burdens in the cardiovascular morbidity with complication subgroup. This is likely an underestimating of actual psychological burdens since patients could develop mental health conditions like depression and anxiety disorders, substance use disorders over the course of TB treatment (23). Our findings highlighted psychological support, active case finding, and early intervention of mental health disorders.

Despite the difference in overall costs, the share of OOP expenditure was much lower in subgroups that incurred more overall expenditure. We found that inpatient services as well as the number of chronic conditions may be the primary driver of expenditures. Since the reimbursement rates tend to be more generous for hospitalization than outpatient services, the OOP expenditures have been offset by insurance reimbursement, which could have a positive impact on care continuation and relieving financial burdens that patients face. However, the one-size-fits-all approach of the current reimbursement schemes oftentimes fails to account for the disparate needs of patients with varying chronic condition profiles. Recent health services research on catastrophic expenditure among patients with TB from the Ningbo region found that 37.1% of TB patients and their households faced catastrophic economic risks (24), therefore, reimbursement rates should be tailored to provide additional support for financially vulnerable patients with greater healthcare needs.

Our study has several limitations. First, we relied on recorded administrative data to identify chronic conditions, which are subject to coding errors and potentially, misclassification bias. Second, our findings may not be generalizable to TB patients in other settings, such as those in HIV endemic countries. Third, our estimates on healthcare expenditure adopted a healthcare system and patients' perspective, which could be different from a payers' perspective. Lastly, there are unmeasured confounding factors that are due to the inherent limitations of our retrospective design, preclude us from establishing a causal relationship between chronic comorbidity combinations and meaningful clinical outcomes, healthcare use, and expenditure.

## Conclusion

In conclusion, the results of the current study offered much-needed evidence on comorbidity patterns in TB patients from a real-world perspective. The distinct patterns of multimorbidity highlight opportunities to better personalize TB care for patients with pre-existing conditions and a tailored, multidisciplinary approach to managing an increasing older TB population facing multifaceted health requirements.

## Data Availability Statement

The raw data supporting the conclusions of this article will be made available by the authors, without undue reservation.

## Ethics Statement

The studies involving human participants were reviewed and approved by Ningbo Municipal Center for Disease Control and Prevention. Written informed consent for participation was not required for this study in accordance with the national legislation and the institutional requirements.

## Author Contributions

QC, YC, and JZ designed the study. QC, YC, YX, and FJ were primarily responsible for data analysis and writing the article. JZ and TY contributed critically to the writing of the article. All authors contributed to the article and approved the submitted version.

## Funding

This work was supported by the Zhejiang Medical Research Project (2018KY733), the Natural Science Foundation of Ningbo (2019A610386 and 2019A610385), and Ningbo Medical and Health Brand Discipline (PPXK2018-10).

## Author Disclaimer

The content is solely the responsibility of the authors and does not necessarily represent the official views of the funding agencies.

## Conflict of Interest

The authors declare that the research was conducted in the absence of any commercial or financial relationships that could be construed as a potential conflict of interest.

## Publisher's Note

All claims expressed in this article are solely those of the authors and do not necessarily represent those of their affiliated organizations, or those of the publisher, the editors and the reviewers. Any product that may be evaluated in this article, or claim that may be made by its manufacturer, is not guaranteed or endorsed by the publisher.

## References

[1] WHO. Global Tuberculosis Report, 2020. (2020). Available online at: https://apps.who.int/iris/bitstream/handle/10665/336069/9789240013131-eng.pdf (accessed September 16, 2021).

[2] The Academy of Medical Sciences, 2018 (2018). Available online at: https://acmedsci.ac.uk/file-download/82222577 (accessed September 16, 2021).

[3] Hou J Wang G Wang F Cheng J Ren H Zhuang H. Guideline of prevention and treatment for chronic hepatitis B (2015 update). J Clin Transl Hepatol. (2017) 5:297–318. 10.14218/JCTH.2016.00019 29226097 PMC5719188

[4] Gao Y Liu M Chen Y Shi S Geng J Tian J. Association between tuberculosis and COVID-19 severity and mortality: a rapid systematic review and meta-analysis. J Med Virol. (2021) 93:194–6. 10.1002/jmv.26311 32687228 PMC7405273

[5] Reis-Santos B Gomes T Macedo LR Horta BL Riley LW Maciel EL. Prevalence and patterns of multimorbidity among tuberculosis patients in Brazil: a cross-sectional study. Int J Equity Health. (2013) 12:61. 10.1186/1475-9276-12-61 23962018 PMC3765118

[6] Fortin M Stewart M Poitras ME Almirall J Maddocks H. A systematic review of prevalence studies on multimorbidity: toward a more uniform methodology. Ann Fam Med. (2012) 10:142–51. 10.1370/afm.1337 22412006 PMC3315131

[7] von Elm E Altman DG Egger M Pocock SJ Gøtzsche PC Vandenbroucke JP. Strengthening the Reporting of Observational Studies in Epidemiology (STROBE) statement: guidelines for reporting observational studies. BMJ. (2007) 335:806–8. 10.1136/bmj.39335.541782.AD 17947786 PMC2034723

[8] Healthcare Cost and Utilization Project (HCUP). User Support (HCUP-US). (2021). Available online at: https://www.ahrq.gov/cpi/about/otherwebsites/hcupnet.ahrq.gov/index.html (accessed September 16, 2021).

[9] Chronic Condition Indicator (CCI) for ICD-10-CM (Beta Version). (2021). Available online at: https://www.hcup-us.ahrq.gov/toolssoftware/chronic_icd10/chronic_icd10.jsp (accessed September 16, 2021).

[10] Rosellini AJ Szentkúti P Horváth-Puhó E Smith ML Galatzer-Levy I Lash TL. Latent classes of posttraumatic psychiatric comorbidity in the general population. J Psychiatr Res. (2021) 136:334–42. 10.1016/j.jpsychires.2021.02.013 33636689 PMC8485142

[11] Yang BR Um HY Lee MT Kim MS Jung SY. Characterizing tramadol users with potentially inappropriate co-medications: a latent class analysis among older adults. PloS ONE. (2021) 16:e0246426. 10.1371/journal.pone.0246426 33606722 PMC7894862

[12] Zhou J Nutescu EA Han J Calip GS. Clinical trajectories, healthcare resource use, and costs of long-term hematopoietic stem cell transplantation survivors: a latent class analysis. J Cancer Survivor Res Pract. (2020) 14:294–304. 10.1007/s11764-019-00842-1 31897877 PMC7390707

[13] Schreiber JB. Latent class analysis: an example for reporting results. Res Soc Administr Pharm. (2017) 13:1196–201. 10.1016/j.sapharm.2016.11.011 27955976

[14] Manning WG Mullahy J. Estimating log models: to transform or not to transform? J Health Econ. (2001) 20:461–94. 10.1016/S0167-6296(01)00086-8 11469231

[15] Pregibon D. Goodness of link tests for generalized linear models. J R Stati Soci Ser C. (1980) 29:15–24. 10.2307/2346405

[16] Belotti F Deb P Manning WG Norton EC. twopm: two-part models. Stata J. (2015) 15:3–20. 10.1177/1536867X1501500102

[17] Pan Y Chen S Chen M Zhang P Long Q Xiang L. Disparity in reimbursement for tuberculosis care among different health insurance schemes: evidence from three counties in central China. Infect Dis Poverty. (2016) 5:7. 10.1186/s40249-016-0102-4 26812914 PMC4729161

[18] Yuan B Li J Wu L Wang Z. Multi-level social health insurance system in the age of frequent employment change: the urban unemployment-induced insurance transition and healthcare utilization in China. Healthcare. (2019) 7:77. 10.3390/healthcare7020077 31200482 PMC6627781

[19] Nielsen AM Kent P Hestbaek L Vach W Kongsted A. Identifying subgroups of patients using latent class analysis: should we use a single-stage or a two-stage approach? A methodological study using a cohort of patients with low back pain. BMC Musculoskelet Disord. (2017) 18:57. 10.1186/s12891-017-1411-x 28143458 PMC5286735

[20] Omar N Wong J Thu K Alikhan MF Chaw L. Prevalence and associated factors of diabetes mellitus among tuberculosis patients in Brunei Darussalam: a 6-year retrospective cohort study. Int J Infect Dis. (2021) 105:267–73. 10.1016/j.ijid.2021.02.064 33610780

[21] Araia ZZ Mesfin AB Mebrahtu AH Tewelde AG Osman R Tuumzghi HA. Diabetes mellitus and its associated factors in tuberculosis patients in maekel region, eritrea: analytical cross-sectional study. Diabetes Metab Synd Obes Targets Ther. (2021) 14:515–23. 10.2147/DMSO.S293557 33568928 PMC7869713

[22] Kang W Du J Yang S Yu J Chen H Liu J. The prevalence and risks of major comorbidities among inpatients with pulmonary tuberculosis in China from a gender and age perspective: a large-scale multicenter observational study. Eur J Clin Microbiol Infect Dis. (2021) 40:787–800. 10.1007/s10096-020-04077-2 33094354

[23] Lee G Scuffell J Galea JT Shin SS Magill E Jaramillo E. Impact of mental disorders on active TB treatment outcomes: a systematic review and meta-analysis. Int J Tuberculosis Lung Dis. (2020) 24:1279–84. 10.5588/ijtld.20.0458 33317672 PMC7740071

[24] Yang T Chen T Che Y Chen Q Bo D. Factors associated with catastrophic total costs due to tuberculosis under a designated hospital service model: a cross-sectional study in China. BMC Public Health. (2020) 20:1009. 10.1186/s12889-020-09136-z 32586305 PMC7318445

