# Supplementary material for: Impact of Multimorbidity Subgroups on the Health Care Use and Clinical Outcomes of Patients With Tuberculosis: A Population-Based Cohort Analysis
Source: Front Public Health. 2021 Oct 8;9:756717. doi: 10.3389/fpubh.2021.756717 (PMC8531479; doi:10.3389/fpubh.2021.756717)
Supplement: Supplementary file 1 [file Table_1.docx]

**Title:**

Impact of multimorbidity subgroups on the health care use, expenditures, and clinical outcomes among patients with tuberculosis: A population-based cohort analysis

**List of Supplemental Tables**

**Supplemental Table 1:** Treatment success and association with chronic condition groups

**Supplemental Table 2**: Univariate summary of **overall** healthcare expenditure according to chronic condition groups (in 2014 CNY terms, adjusted for inflation rates)

**Supplemental Table 3**: Univariate summary of average, individual monthly healthcare expenditure according to chronic condition groups, in 2014 CNY.

**Supplemental Table 4**: Association of chronic condition groups with healthcare expenditure

**Supplemental Table 1:** Treatment success and association with chronic condition groups

| **Chronic condition groups** | OR | 95%CI | | *P*-value |
| --- | --- | --- | --- | --- |
| **Base model*** |  |  |  |  |
| - **No chronic conditions** | Reference | | | |
| - **Respiratory morbidity** | 0.80 | 0.62 | 1.03 | 0.0884 |
| - **Cardiovascular morbidity with complications** | 0.59 | 0.46 | 0.77 | <.0001 |
| - **Cardiovascular morbidity without complications** | 1.27 | 1.10 | 1.47 | 0.0014 |
| - **General morbidity** | 1.12 | 0.98 | 1.28 | 0.0985 |
| **Extended model*** |  |  |  |  |
| - **No chronic conditions** | Reference | | | |
| - **Respiratory morbidity** | 0.68 | 0.49 | 0.93 | 0.0163 |
| - **Cardiovascular morbidity with complications** | 0.59 | 0.42 | 0.83 | 0.0027 |
| - **Cardiovascular morbidity without complications** | 1.40 | 1.15 | 1.71 | 0.0008 |
| - **General morbidity** | 1.18 | 0.97 | 1.43 | 0.0903 |

*Adjusted for age groups and sex.

**Adjusted for age groups, sex, type of health insurance, tuberculosis retreatment status, smoking and alcohol consumption patterns.

Abbreviations: OR, odds ratio; CI, confidence interval.

**Supplemental Table 2**: Univariate summary of overall healthcare expenditure according to chronic condition groups (in 2014 CNY, adjusted for inflation rates)

| Characteristics | Overall | | No chronic conditions | | Respiratory morbidity | | Cardiovascular morbidity with complications | | Cardiovascular morbidity without complications | | General morbidity | |
| --- | --- | --- | --- | --- | --- | --- | --- | --- | --- | --- | --- | --- |
| **Inpatient expenditure** |  |  |  |  |  |  |  |  |  |  |  |  |
| Mean, SD | 4881 | 19208 | 2069 | 12310 | 9099 | 34725 | 12153 | 39559 | 4681 | 16806 | 3889 | 14764 |
| Median (IQR) | 0 | 0-2456 | 0 | 0-0 | 0 | 0-8027 | 0 | 0-9119 | 0 | 0-3240 | 0 | 0-0 |
| Skewness | 12 |  | 28 |  | 11 |  | 7 |  | 9 |  | 10 |  |
| **Outpatient expenditure** |  |  |  |  |  |  |  |  |  |  |  |  |
| Mean, SD | 3675 | 4675 | 1989 | 2470 | 3369 | 3953 | 5139 | 6572 | 4070 | 5484 | 3324 | 3868 |
| Median (IQR) | 2576 | 439-5081 | 1166 | 0-3068 | 2164 | 145-5105 | 3530 | 95-7012 | 2929 | 673-5385 | 2306 | 392-4790 |
| Skewness | 5 |  | 2 |  | 2 |  | 3 |  | 6 |  | 3 |  |
| **Pharmacy expenditure** |  |  |  |  |  |  |  |  |  |  |  |  |
| Mean, SD | 1495 | 2379 | 634 | 1052 | 1530 | 2096 | 2547 | 3906 | 1783 | 2698 | 1212 | 1915 |
| Median (IQR) | 779 | 91-1933 | 217 | 0-849 | 676 | 16-2286 | 1228 | 44-3531 | 1059 | 186-2363 | 606 | 64-1578 |
| Skewness | 5 |  | 4 |  | 2 |  | 4 |  | 5 |  | 6 |  |
| **Overall expenditure** |  |  |  |  |  |  |  |  |  |  |  |  |
| Mean, SD | 10052 | 20976 | 4691 | 13032 | 13998 | 35873 | 19839 | 40835 | 10533 | 19232 | 8425 | 16411 |
| Median (IQR) | 4911 | 1106-11471 | 1951 | 16-5594 | 6151 | 716-14915 | 10189 | 1761-22285 | 5684 | 1488-12428 | 4177 | 923-9633 |
| Skewness | 10 |  | 24 |  | 11 |  | 7 |  | 7 |  | 8 |  |
| **Out-of-pocket expenditure** |  |  |  |  |  |  |  |  |  |  |  |  |
| Mean, SD | 2243 | 6638 | 1695 | 6898 | 3231 | 10614 | 4169 | 11488 | 2231 | 6563 | 1968 | 5373 |
| Median (IQR) | 448 | 6-1667 | 153 | 0-1136 | 586 | 8-2990 | 879 | 16-2919 | 399 | 8-1639 | 448 | 5-1505 |
| Skewness | 10 |  | 27 |  | 11 |  | 6 |  | 8 |  | 9 |  |

Abbreviations: SD, standard deviation; IQR, interquartile range.

**Supplemental Table 3**: Univariate summary of average, individual monthly healthcare expenditure according to chronic condition groups, in 2014 CNY.

| Characteristics  Mean, SD | No chronic conditions | | Patients with multimorbidity | | Respiratory morbidity | | Cardiovascular morbidity with complications | | Cardiovascular morbidity without complications | | General morbidity | |
| --- | --- | --- | --- | --- | --- | --- | --- | --- | --- | --- | --- | --- |
| Sample size | 5924 | | 3727 | | 220 | | 185 | | 1292 | | 2030 | |
| **Inpatient expenditure** | 321 | 2444 | 788 | 3936 | 1459 | 4740 | 1738 | 4439 | 653 | 2518 | 715 | 4479 |
| **Outpatient expenditure** | 246 | 349 | 454 | 598 | 450 | 555 | 581 | 687 | 487 | 582 | 422 | 601 |
| **Pharmacy expenditure** | 76 | 127 | 181 | 286 | 207 | 360 | 277 | 408 | 211 | 295 | 150 | 252 |
| **Overall expenditure** | 643 | 2532 | 1423 | 4106 | 2116 | 4951 | 2595 | 4541 | 1352 | 2722 | 1286 | 4640 |
| **Out-of-pocket expenditure** | 237 | 1569 | 336 | 1245 | 531 | 1719 | 519 | 1225 | 307 | 1005 | 316 | 1319 |
| **Proportion of out-of-pocket costs relative to overall expenditures** | 36.9% | | 23.6% | | 25.1% | | 20.0% | | 22.7% | | 24.5% | |

Abbreviations: SD, standard deviation.

**Supplemental Table 4**: Association of chronic condition groups with healthcare expenditure

| **Chronic condition groups** | **Conditional incremental effects** | **95%CI** | | **P-value** |
| --- | --- | --- | --- | --- |
| **Overall costs** |  |  | |  |
| **Base model*** |  |  |  |  |
| - **No chronic conditions** | Reference |  |  |  |
| - **Respiratory morbidity** | 21093 | 17466 | 25474 | <.0001 |
| - **Cardiovascular morbidity with complications** | 10806 | 10072 | 11593 | <.0001 |
| - **Cardiovascular morbidity without complications** | 10019 | 9476 | 10593 | <.0001 |
| - **General morbidity** | 6851 | 6575 | 7139 | <.0001 |
| **Extended model*** |  |  |  |  |
| - **No chronic conditions** | Reference |  |  |  |
| - **Respiratory morbidity** | 16360 | 12615 | 21215 | <.0001 |
| - **Cardiovascular morbidity with complications** | 10987 | 9542 | 12651 | <.0001 |
| - **Cardiovascular morbidity without complications** | 10279 | 8999 | 11741 | <.0001 |
| - **General morbidity** | 7832 | 6928 | 8853 | <.0001 |
| **Out-of-pocket costs** |  |  |  |  |
| **Base model*** |  |  |  |  |
| **No chronic conditions** | Reference |  |  |  |
| **Respiratory morbidity** | 4620 | 3600 | 5928 | <.0001 |
| **Cardiovascular morbidity with complications** | 2524 | 2286 | 2787 | <.0001 |
| **Cardiovascular morbidity without complications** | 2605 | 2410 | 2816 | <.0001 |
| **General morbidity** | 2807 | 2649 | 2974 | <.0001 |
| **Extended model*** |  |  |  |  |
| **No chronic conditions** | Reference |  |  |  |
| **Respiratory morbidity** | 4543 | 3163 | 6525 | <.0001 |
| **Cardiovascular morbidity with complications** | 2583 | 2110 | 3163 | <.0001 |
| **Cardiovascular morbidity without complications** | 2817 | 2318 | 3424 | <.0001 |
| **General morbidity** | 3136 | 2632 | 3736 | <.0001 |

Abbreviations: OR, odds ratio; CI, confidence interval

*Adjusted for age groups and sex.

** Adjusted for age groups, sex, type of health insurance, tuberculosis retreatment status, smoking, alcohol consumption patterns, and number of hospital admissions at baseline.
